# Breaking up with my idol: A qualitative study of the psychological adaptation process of renouncing fanship

**DOI:** 10.3389/fpsyg.2022.1030470

**Published:** 2022-12-16

**Authors:** Yiqing He, Ying Sun

**Affiliations:** School of Education, Tianjin University, Tianjin, China

**Keywords:** fans, idol worship, renouncement, breakup, psychological adaptation, loveshock, netnography

## Abstract

**Introduction:**

This study aimed to explore the psychological adaptation process of renouncing fanship due to para-loveshock in the context of fandom culture.

**Methods:**

We adopted netnography to explore social media platforms used by fans in China (Weibo, WeChat, and Douban) as research fields for 3 years.

**Results:**

(1) The process of “breaking up with” or renouncing an idol can be divided into three phases: the resistance phase with acute stress, the negotiation phase with bargaining, and the recovery phase with attachment reconstruction. In the resistance phase, fans displayed acute stress responses due to loveshock in psychological, physical, and behavioral aspects. In the negotiation phase, fans faced four barriers to renouncement: cognitive dissonance, emotional attachment, behavioral dependence, and social threat. They bargained within the three types of cognitive framework before deciding to “leave” or “re-follow” their idol. In the recovery phase, fans adopted two types of strategies to promote recovery: healing the past and facing the future. Healing the past involved public outcry, sharing their breakup plans, cognitive reconstruction, and seeking closure to the fan role. Facing the future involved switching environments, seeking new interests, and inhibiting the re-intrusion of trauma cues. (2) Internal factors affecting the psychological adaptation process of renouncement include the level of initiative, attribution styles, experience, attachment status and core belief systems, and alternative lifestyles; external factors include social support, peer pressure from the fan community, life stressors, and types and impact of traumatic events. (3) Based on the two dimensions of orientation and commitment, fans were classified into four types: short-term rational, short-term passionate, bounded loyal, and unconditionally loyal, corresponding to non-traumatic, stressful, accumulated, and traumatic breakup processes, respectively. (4) The post-renouncement growth of fans mainly manifested in the development of mental modes, coping skills toward trauma, emotional adaptation experience, and behavior patterns.

**Implications:**

This investigation of the recovery process from para-loveshock after renouncement of fanship can provide theoretical and practical insights into the development of psychological resilience for fans, reduction of the psychological distress and negative outcomes, and public governance on social media platform and cyber pop culture industry.

## Introduction

According to Sina Weibo, in 2019, the registered fans of entertainment stars made more 19.2 billion site visits in China, with an annual increase of 2.5 billion. In 2020, the scale of China’s idol market exceeded 100 billion yuan, of which the income from fans’ consumption was about 50 billion yuan. Since 2020, the mass media in China has published articles arguing that pop star fandom or idol worship subculture based on social media has had significant negative impacts on the lives of fans and their families, as well as Chinese society as a whole (Yin, 2020; Zhang and Negus, 2020). This media focus has attracted the attention of people calling for governance and intervention on negative outcomes of fandom culture (Yin, 2021).

Celebrity worship is associated with extensive stressful outcomes and may lead to traumatic breakup experiences for fans in three ways (McCutcheon et al., 2021). First, the negative events and excessive idolatrous behavior in fandom (e.g., social media addiction, inter- and intra-group conflict, cyber-violence, and forced purchases) can induce a variety of negative emotions and behavior in fans, causing damage to fans’ study, work, and interpersonal relationships, and even triggering dangerous behavior such as self-harm or suicide attempts (Liu et al., 2022). Second, it was found that celebrity worship occurs more often in younger individuals (adolescents or young adults; Maltby et al., 2005; Zsila et al., 2019). Younger fans may be experiencing a critical stage of the separation-individuation process (Mattanah et al., 2004), and are considered more at risk of suffering from breakup shock, because they often lack impulse control, social resources, and coping skills when encountering negative events. This can result in excessive stress, anxiety, psychological distress, and risk-taking behaviors (Cheung and Yue, 2019). Third, some fans perceive the idol as an intimate romantic partner (McCutcheon et al., 2002), or become attached to the idol in para-social relationship (Collins and Van Dulmen, 2006; Cheung and Yue, 2011). These fans tend to show high levels of attachment anxiety, doubt their self-worth, and be susceptible to negative judgment and information relating to their idols (Stevens and Hamann, 2012; Meyers-Levy and Loken, 2015).

As celebrity worship is becoming increasingly popular today (Giles and Maltby, 2004), it would be beneficial to explore the breakup experience and adaptation process of fans, help them reduce physical responses and psychological distress, and promote recovery from para-loveshock (Smith et al., 2011). Therefore, our research question was, *How do fans go through the psychological adaptation process of renouncing fanship?* By reviewing existing literature on celebrity and idol worship, it was found that few studies showed concerns for the breakup stage, that is, how fans leave their idols. To fill the research gap, we referred to two streams of literature for theoretical perspectives from similar context: (1) dissociation of intimate/romantic relationship, grief of loss, and trauma recovery; and (2) the withdrawal from behavioral addiction. By referring to these connected studies, similarities and differences between the contexts were compared and identified to expand the theoretical boundaries of idol worship literature.

This study conducted a 3-year netnography, collected primary and secondary qualitative data from multiple sources on social media platforms, and examined the phases of adaptation process, factors related to the process, breakup style, and the post-breakup growth outcome. The present study contributes to the existing literature in three ways. First, it adds to the literature of celebrity worship by focusing on the breakup stage to help fans finalize their worship behavior. Second, this study applies the existing theory of relationship breakdown, grief and trauma to the context of idol worship to facilitate recovery and improve psychological resilience of fans. Third, it contributes from the perspective of behavioral addiction, which is applied to the idol worship context to help fans cope with mental and physical withdrawal symptoms. By referring to the distant literature, the theoretical boundary of celebrity worship literature was expanded. In terms of practical implications, this study aims to help fans recover from para-loveshock, reduce psychological distress and negative outcomes, and improve their psychological resilience. Moreover, it provides insights into public governance on social media platforms and the cyber pop culture industry.

## Literature review and theoretical foundation

### Idol worship and renouncement of fanship

The word “Fan” originated from “fanatic” which is associated with religious zealotry and refers to someone who is loyal, enthusiastic, and an ardent admirer of some interest (McCutcheon et al., 2002). Idol worship traditionally means the worship of a representation of God, religious figure or an enchanted figure, which is broader than celebrity worship. Celebrity worship was defined as the increased admiration or an obsessive preoccupation toward a famous media figure (Brooks, 2021). In the context of pop culture, the idol or celebrity usually refers to pop star, sports hero, etc. Within the past 20 years, fandom, which refers to the community of fans who are attached to each other with common ingroup identity, has been evolving substantially with the development of social media platform (Laffan, 2021).

Previous studies on celebrity worship and idol worship within the context of pop culture discussed the intrinsic nature of this phenomenon (Till, 2010; Gleason et al., 2017); examined behavioral and psychological features of online and offline fans (Williams and Ho, 2016; Kunert, 2021), especially the psychological well-being (Brooks, 2021), psychiatric symptoms (e.g., depression, anxiety, difficulties in integration of experiences in various life domains, and maladaptive daydreaming; Somer et al., 2017; Zsila et al., 2018), and pathological, addictive, or deviant behavior (McCutcheon et al., 2002; Zsila et al., 2019) associated with entertainment idols within a clinical context (Maltby et al., 2006; Sheridan et al., 2007); developed multiple measurement tools (Maltby et al., 2006; Cheung and Yue, 2011); identified the correlates, antecedents and outcomes of celebrity worship (Ang and Chan, 2018; McCutcheon et al., 2021), including demographics (Gleason et al., 2017), personality (Maltby and Day, 2017), social, behavioral, and psychological factors (Brooks, 2021; Laffan, 2021); explored the operating mechanisms of fandom and fan culture within the field of pop culture and social media platforms (Van den Bulck et al., 2016; Yin, 2020), the routine practices of “algorithmic culture” (Alaimo and Kallinikos, 2017; Fung, 2019; Livingstone, 2019), the sense of duty and obligation as commitment to the community and the idol (Zhang and Fung, 2017), as well as the interaction and co-evolution among multiple actors including the idol, pop industry, fans, and social media platform (Giles, 2002; Yan and Yang, 2021). However, few of them have focused on the final stage of idol worship, including the breakup and recovery process.

Fanship was conceptualized to reflect the personal identity of one’s psychological attachment to their interest (Reysen and Lloyd, 2012; Stephens et al., 2021). It was suggested that fanship is a fluctuating phenomenon that shows a certain lifecycle (van Driel et al., 2022), indicating that one’s fanship intensity changes over time (Schroy et al., 2016; Laffan, 2021). Learning how to adjust to the end of a relationship is a key task of emerging adulthood (O’Sullivan et al., 2019). Renouncing fanship (i.e., breaking up with the idol) can be interpreted as a state or process, where a person no longer considers their identity as someone’s fan. In this study, the process of breaking up can be defined as the gradual change from a state of deep attachment, obsession, identification, and commitment to the idol and the fan community as a loyal fan to a non-fan (Rezapour et al., 2021). The breakup can be triggered by the idols, the fan community, or the fans themselves (Bravo et al., 2017). Trigger by the idol refers to negative events of the idol, for example, public exposure of a secret partner, infidelity, and engaging in criminal acts. Trigger by the community refers to negative events that occur in the fandom, such as fandom conflicts, compulsive fundraising, or cyber violence. Fans may also leave the idol for personal reasons such as fading passions and attachments, or life stressors. Renouncing fanship can be a stressful process. When the attempt fails, fans “return” and resume fanship to the idol.

### Dissociation of relationship, grief of loss, and trauma recovery

We not only reviewed the related literature on celebrity worship, but also borrowed a theoretical lens from similar contexts, including dissociation of intimate/romantic relationship, grief of loss, and trauma recovery. It was indicated that an intense emotional attachment with a celebrity may serve as compensation for social relationships (Giles, 2017; Brooks, 2021). The development of social media has substantially enhanced such parasocial relationships between fans and celebrities with more intimate dynamics (Baym, 2018; Hopkins, 2019), such as imaginary romantic relationship or kinship which is similar to real life (Chung and Cho, 2017; Stever, 2017). These certain types of intimate parasocial relationships with deep obligation and commitment to the celebrity are shared in East Asia (Chen, 2017). Therefore, it is necessary to review the literature on breakup, grief of loss, and trauma recovery to enlighten this study (Michael and Cooper, 2013).

Romantic breakup is considered one of the most distressing, traumatic, and stressful experiences in youth (Bravo et al., 2017), and accompanied by feelings of panic, fear, and helplessness. It may be followed by positive outcomes, such as post-breakup growth, or adverse outcomes that persist for many months, such as anxiety, depression, grief (Earp et al., 2017), maladaptive coping, suicidal tendencies (Bravo et al., 2017; Palacio-González et al., 2017). Both biological and psychological vulnerabilities contribute to emotional disorders (Rezapour et al., 2021). Rumination is associated with poor adjustment (Wrape et al., 2016). Entering a new relationship, having strong parental and peer support, self-esteem, grit, and optimism are associated with less distress and better psychological adjustment (O’Sullivan et al., 2019; Oliffe et al., 2022). The distress following breakup subsides as time goes by in most cases (Belu et al., 2016).

The four-stage theory of grief and loss included: shock-numbness, yearning-searching, disorganization-despair, and reorganization (Parkes, 2002; Obst et al., 2020). The five-stage model included: denial-dissociation-isolation, anger, bargaining, depression, and acceptance (Kübler-Ross and Kessler, 2005; Valliani and Mughal, 2022). These stage theories of grief have been adopted in a wide variety of losses including separation, divorce, death, and other forms of breakup (Prigerson et al., 2021). Studies that followed indicated that some stages were not inevitable (e.g., depression) and proposed a stage model of numbness-disbelief, separation distress (yearning-anger-anxiety), depression-mourning, and recovery (Jacobs, 1993). The stress response syndrome was also depicted in phases: outcry, denial, intrusion, working through, and completion (Horowitz, 1990; Easton, 2013). There were also other proposed models with differentiated phases including initial denial and avoidance of the loss, alarm reactions (restlessness and physiological complaints), distress, inquisition, confirmation, seeking comfort, cognitive restructuring, psychological reintegration or reorganization, acceptance, reinvestment in other relationships and life pursuits, adaptation to loss, and psychospiritual transformation. (Rothaupt and Becker, 2007; McCoyd et al., 2021). The previous theoretical foundation enlightens our study on delineating the process of renouncing fanship into phases.

Breakup with an idol can turn into a traumatic event. In this case, the risk factors of trauma recovery may overlap with the key factors influencing fans’ para-loveshock recovery. In terms of trauma recovery, the four types of risk factors associated with stressful trauma recovery were reviewed. The first type is demographics including age, gender, education, IQ, and ethnicity (Joormann et al., 2022). The second type includes pre-trauma correlates such as prior psychopathology, prior trauma exposure, familial psychiatric history, and neurobiological factors (Rosellini et al., 2018). The third type concerns peri-trauma factors including the duration/severity of trauma exposure, core belief challenge, and subjective fear (Oaie et al., 2018; Meiser-Stedman et al., 2019; Beaudoin et al., 2021). The fourth type involves post-trauma factors including coping strategies, rumination, resilience, attribution style, access to needed resources, social support, specific cognitive patterns, gratitude, etc. (Trickey et al., 2012; Sayed et al., 2015).

### Behavioral addiction

The perspective of behavioral addiction in the literature of celebrity worship sheds some light on our study’s goal to explore the final breakup and recovery stage of fans. Based on the widely adopted conceptual model of absorption-addiction idolatry (Maltby et al., 2006; Cheung and Yue, 2019), the absorption with a celebrity can be described with three dimensions: the entertainment-social dimension which is usually benign, intense-personal dimension which reflects compulsive feelings, and borderline-pathological dimension which represents the most extreme expression of celebrity worship (McCutcheon et al., 2002). The second and third levels were found to be associated with problematic behavior (McCutcheon et al., 2021). Celebrity worship is found to be associated with excessive and addicted behaviors in several studies, such as problematic Internet and social media use, forced purchases, and love addiction (Zsila et al., 2018, 2021), “dependent love” and “game-playing” love (McCutcheon et al., 2016). It has been proposed that the motivational forces of psychological absorption in celebrity worshiper resemble those of addiction (McCutcheon et al., 2002).

According to the broad view of love addiction (or pathological love), romantic relationship is considered a social attachment on the spectrum of natural addictive motivation that engages the brain’s reward system (Fisher et al., 2016). Love addiction showed overlapping mechanisms with other behavioral addictions on the cognitive level and neurochemical level. It was found of higher rate in the population of young students and was defined as a behavioral pattern that exhibits excessive and maladaptive interest toward romantic partners, leading to multiple negative consequences such as loss of impulse control, low self-worth, or craving (Sanches and John, 2019). When faced with the loss of love, the stress and withdrawal symptoms, such as anxiety and depression, would drive one to reinstate the relationship or quickly move on to a new partner (Redcay and Simonetti, 2018).

Preventions and interventions are proposed to help withdraw from behavioral addiction, for example, avoiding continuous usage or carrying out short-term abstinence (Zhou et al., 2021), turning to other interests, improving offline social activities and communication for real-life social support (Meshi and Ellithorpe, 2021), maintaining daily physical exercises, sticking to regular circadian rhythms (Mérelle et al., 2017; Kwok et al., 2021), etc. By the approach of cognitive reconstruction, one would realize the negative consequences of addictive behavior (Hou et al., 2019).

### The current study

By reviewing existing literature on celebrity worship and idol worship within the context of pop culture, it was found that few of them showed concerns for the final stage of idolatry, that is, how they leave their idol. However, previous studies on breakup, grief, trauma recovery and behavioral addiction provided extensive theoretical foundations. As a result, our research objective is to explore the psychological adaptation process of how fans renounce fanship. To be more specific, this study aims to describe the breakup process in phases, identify key factors influencing the process, categorize the breakup style, and generalize the post-breakup growth. By doing this, we not only offer a clear picture of how fans renounce fanship, i.e., how fans break up with the idol, but also clarify individual differences to outline theoretical boundaries and set up a positive hope for fans to pursue at the end of a stressful breakup. This research helps reduce the psychological distress and negative outcomes in fans and helps improve their psychological resilience.

## Materials and methods

### Netnography research of online subcultures

Netnography is an interpretive approach that adapts traditional ethnographic techniques to cyberspace and Internet culture (Kozinets, 2002). It has also been defined as ethnographic research conducted in a virtual environment *via* and for the Internet or using different data collection tools in a virtual environment on the Internet. It is believed that such long-term, immersive online participatory observation allows the researcher access to the inner vision of the culture holders (Ashman et al., 2021). Netnographic studies in China began in the last 20 years and have shown a rapid rise with the prevalence of mobile social media platforms. Most of these studies have focused on the topic of cyber subculture and identity, of which fan communities draw the most attention. Meanwhile, WeChat and Weibo have been the most investigated social media platforms in China (Hou et al., 2018). Many researchers emphasize the importance of “immersion” in the online field, which mainly refers to sustained mental focus, virtual presence in the community, deep commitment for a long enough period or at a high enough frequency, and falling into a state of absorbed attention to understand the feelings of the research subject. It was proposed that researchers need to act as “insiders” within the fan community and become “aca-fans” (Jenkins, 2012). The first author of this study sought to become an insider as one of the most loyal fans and a resident of the community to experience the internal lifestyle and culture. According to Fitzsimons (2013), the author’s experience of “immersion” goes through roughly three stages, the first of which is separation, that is, crossing over from the real world into the virtual world and becoming a “new fan.” The second stage is conversion, that is, learning fans’ skills and behaviors, and establishing an identity as a legitimate fan identified by the community. The third stage is transformation, indicating immersion into and obsession with the virtual identity, and the prolonged participation in the community as a real fan. As a researcher, it is also necessary to keep the ability to step out of the role when collecting and analyzing the data from a rational and objective perspective as an “outsider.”

### Research field and data collection

This study adopted the netnographic approach to observe fan behavior within online communities in depth. We selected a newly debuted young pop star in China whose fan community was well-organized on social media and adopted this fan community as the research object. We collected information not only about this community, but also about other celebrity fans through fan networks to verify and replicate the research findings and enhance the validity of the study. We conducted the research between July 2018 and December 2021. This study used social media platforms such as Weibo, WeChat, and Douban (the most widely used social media apps in China) as online fields, with Weibo being the main field. The first author used three Weibo accounts for multiple identities and purposes to participate in the fan community for interaction, observation, messaging, fan support activities (e.g., fundraising, video editing). Data collected mainly included fan community chat transcripts, posts, and interactions on the public platforms, opinions from stakeholders and bystanders (e.g., fans of other pop stars, industry insiders), relevant articles and reports, and relevant news of the selected pop star. Information about the same event was collected from multiple sources to perform triangulation for validation. The text sources were coded as “source-ID-time.”^1^ The information for the main online communities we observed and participated in is shown in Table 1. A combination of convenience sampling and purposive sampling (age and occupation) were used to select eight fans who had already quit fandom for in-depth interviews, mainly to understand their physical and psychological experiences and behaviors during the breakup process. The information of the eight fans is shown in Table 2. The findings were verified and modified in other fan communities to enhance the validity of the study.

**Table 1:** Information of the main online groups observed.

Platform	Coding for group	Scale	Emerging time point	Follow-up period	Affiliated groups (scale)
Weibo	Official support group A1; A2	1,000; 800	Late 2017	2019.8-	
Weibo	Group B	About 60	2018.12	2019.1-	B1 (150), B2 (90), B3 (20)
Weibo	Group C	About 50	Unknown	2019.4-	C1 (70), C2 (15)
WeChat	Group D	About 30	2018.7	2018.8-	D1 (120), D2 (50), D3 (60)
Weibo	Group E	About 10	2019.5	2019.5-	
Weibo	Group F	About 70	2019.7	2019.10-	

**Table 2:** Information of the eight fans selected for follow-up interview.

Number	ID initials	Professions	Age (years old)	Affiliated online community
1	TYT	High school student	16	Group D
2	SMT	Junior college student	20	Groups B, C
3	XBA	Senior college student	22	Core member in Group C
4	HTX	Graduate student	25	Groups B, C
5	EVA	Elementary school teacher	25	Group C
6	SNC	Civil servant	28	Groups C, D
7	DXJ	Manager in advertising service	33	Administrator of Group C, Core member in Group D
8	ADJ	Manager in marketing department	35	Administrator of Group D, Core member in Group C

In recent years, researchers have identified several potential ethical issues with the qualitative data generated and obtained online due to the “traceability” of quotes, including anonymity and pseudonymity, and tensions over public/private space (Roberts, 2015). Therefore, we adopted several strategies to eliminate the potential risk for both the researchers and online fans. First, to protect both the researchers and the online fans observed and interacted with in this study, anonymity was maintained throughout the entire process. Quotes were translated from the raw data in Chinese and some were slightly paraphrased (without changing the meaning), which was the first protective shield to prevent any possibility of public tracing of identities (Hewitt-Taylor and Bond, 2012). We did not include any detailed information in the quotes that might reveal private information. Furthermore, the quotes cited in this article are all typical remarks common in fan communities, which we discovered during our 3-year observation (Markham, 2012; Bond et al., 2013). We double-checked that the quotations were not traceable by using search engines (Malik and Coulson, 2013). We also kept the idols involved in this study anonymous so that their communities would not be identified, thereby protecting the communities and idols themselves. Second, we adopted the approach of previous online ethnographic researchers, keeping our professional and personal identities separate (Paechter, 2013). We took extreme care to ensure that information would not slip between identities.

### Data analysis

Based on the proposed coding strategies (Miles and Huberman, 1994; Eisenhardt and Graebner, 2007), we first cleaned and simplified the raw data, identified the main constructs, and formed a robust causal chain of evidence; then, we conducted discussions among the researchers, verified the conclusion with the research subjects, and adjusted the coding strategy after repeated discussion; finally, we constantly conducted comparisons between the data and the literature, and established a theoretical framework.

To ensure the reliability and validity of this netnographic research, we collected multiple sources of evidence, including chat transcripts within fan groups, private chat transcripts, platform posts and interactions, and opinions from other actors. By immersing ourselves in online group interactions, close relationships with other fans were established, large amount of internal information can be observed. The multiple sources of evidence contributed to triangulation of the conclusion. First, evidence was collected from real time posts and interactions on open platform and internal chat transcripts within fan groups listed in Table 1 to reflect all kinds of reactions from fans experiencing breakup. Subsequently, the private follow-up in-depth interviews, together with the open posts and intra-group chats, helped confirm the sequential order of reactions which lead to the three phases of the adaptation process, and the individual difference of reactions which lead to the categorization of fans and their breakup style. The emerging conclusions were replicated and validated in several cases within and outside the community. Within the community, we followed several rounds of crises during which fans renounce fanship in an intensive way; outside the community, the collapse of other pop stars provided available opportunities to replicate our research. Highly similar reactions and statements implied the saturation of evidence. Moreover, we followed the opinions from stakeholders and bystanders from posts, discussion, articles and reports on Weibo and Douban forum during the outburst of crises to get an external viewpoint. In this case, the loss of fans can be verified from a broader and more objective perspective. Competitive propositions were raised and discussed among the researchers and fans. A robust theoretical framework was developed by repeatedly comparing data and theories to achieve a match.

## Results

### Three phases in the process of renouncing fanship

#### Acute stress – Resistance phase

According to the social cognitive and information processing theories, fans tend to be more sensitive to negative information that cannot be integrated in their core belief systems; for example, they often refuse to accept negative news of their idol and believe that their idol is perfect. Negative and traumatic events with respect to the idol and the fandom lead to heavier stress responses in fans compared with bystanders. During the resistance phase, fans usually show two main behavior tendencies: one is to suppress the traumatic information, (i.e., fans deny or escape from the facts); the other is to deeply process the traumatic information and attempt to obtain more information and clues. For deeply engaged fans, negative events involving idols and fan communities can be regarded as stressful life events intertwined with psychological, cultural, and social aspects. These include primary traumatic events such as idols committing crimes, idols violating public order and morality, privacy exposure, and fights between fan groups, and secondary traumatic events such as negative public opinions and collective online deviant behaviors caused by the primary traumatic events (Shakespeare-Finch, 2011). Successive stressful events can lead to the collapse of fans’ psychological defense mechanisms and trigger acute physiological, psychological, and behavioral responses to stress. In this case, deeply involved fans exhibit psychological crises and deviant behaviors after experiencing traumatic events, which negatively impact their offline lives (Gove, 2018). In this study we describe these acute stress responses during the resistance phase from physiological, psychological, and behavioral aspects.

From a physiological aspect, fans may develop somatization symptoms involving multiple systems such as respiratory, circulatory, digestive, and neurological symptoms alongside emotional problems such as anxiety and depression, which then lead to adaptation problems like poor academic performance and poor peer relationships (Garralda, 2011; Dooley et al., 2015). Life events are the main environmental factors that induce somatization problems (Hart et al., 2013; Bonvanie et al., 2017). Negative events encountered during fanatical idolatry may trigger a variety of stress responses such as chest tightness and chest pain, arrhythmia, difficulty breathing, cold hands and feet, shivering, sleep disturbances, decreased appetite, fatigue, restlessness, sensitivity, startle response, and inability to concentrate:

I cried while shivering… yelling hysterically. (B-db-XMM-21)

Even my heart is hurting. (A-TJX-20)

My hands started shaking even before I finished reading it… (B-wb-XHB-21)

A few days ago, I was too sad to eat, and I couldn’t sleep well at night. I’ve lost a few pounds. (B-wb-FYC-21)

My hands and feet are cold. (B-wb-NLJ-21)

I couldn’t sleep at all at night in that period when my idol was defamed. I was always afraid that he would face cyber-violence again when I fell asleep (and could not protect him). Every day I woke up early and then surfed Weibo. I had a very hard time and even felt there was something wrong with me. (B-wb-XAY-20)

The psychological aspect usually includes both cognitive and emotional components. Cognitive reactions mainly include denial, rumination, selective ignorance, paranoia, and frustration due to cognitive dissonance:

Although I am frustrated tonight… I will definitely not quit fandom. Justice will be late but never absent. (B-wb-21)

I will not give up. I really can’t live without him. (C-SMT-20)

Be calm! The suspicion of a crime is not necessarily a crime! Trust him! (B-wb-21)

I’ve been so blind for the past nine years, and now I deeply regret having liked you. (B-wb-YKF-21)

Emotional reactions mainly include anxiety, depression, anger, fear, sadness, disappointment, shock, agitation, irritability, self-blame, and loneliness, with short-term and long-term negative impacts:

I have liked him for so many years, from high school to grad school. How did this happen? This is so disappointing and sad. (B-wb-ZXA-21)

I didn’t realize it the other day, and I feel a little angry now… taking us for fools? (C-TYT-20)

I turned on my phone and had a look, then I could hear my heart breaking. My heart sank to the bottom, and I felt shocked, angry, sad, aggrieved, and confused, all of which mixed. I wanted to directly quit fandom and revenge. (B-wb-JX-21)

From a behavioral aspect, stressful events trigger insecurity and panic in fans, with increased information-seeking and interactive behaviors. A significant number of discussions about coping strategies were initiated within the fan community. Behavioral responses to stress often include hostile aggression, escaping from reality, self-pity, self-harm, substance abuse, impaired functioning at work and school, and social avoidance, which may result in deviant and extreme behaviors. For example:

I was too scared to look at my phone all night yesterday. (C-JSS-20)

My friend started fasting for [an idol star]. What should I do… she hasn’t eaten since the day before yesterday, and every day she does nothing but sleep, surf the Internet with her mobile phone and weep. (B-db-XMM-21)

Once it’s nighttime I cannot handle it. Bring me the wine. (A-TJX-20)

There are also a lot of self-destructive fans (e.g., committing suicide by taking pills) in the fan community on Weibo. (B-wb-WYS-20)

*Proposition 1A:* The breakup process begins with the resistance phase, during which fans cope with acute stress in physical, psychological, and behavioral ways.

#### Bargaining – Negotiation phase

Fans enter the negotiation phase after the acute stress phase. Information about traumatic events challenges the fans’ original core belief systems, forcing them to undertake cognitive efforts and complete self-adjustment, bringing the core belief system back into balance. In this phase, fans need to overcome four types of obstacles: cognitive dissonance, emotional attachment, behavioral dependence, and social threats.

The most important aspect of the negotiation phase is to adapt cognitive framework within core beliefs and reduce cognitive dissonance. Previous studies have shown that during the intrusive phase of psychological adjustment, gestalt tendencies drive cognitive processing, seeking to integrate the traumatic cues into preexisting mental modes to improve cognitive adaptation and realize post-traumatic growth (Lindstrom et al., 2013). When fans discover that the new information does not match their preexisting mental modes, they attempt to establish new cognitive framework through bargaining. There are three main types of cognitive framework in this phase: (1) They insist on the preexisting mental modes, rejecting new information, placing complete loyalty and trust in their idols and peers, and expressing their determination not to quit fandom:

He didn’t disclose it, and I’ll pretend it didn’t exist… I guess they will break up if they are photographed… Campus relationships don’t last long. (A-DXJ-20)

I'm sure she’ll come back… There’s nothing wrong with her.^2^ (B-wb-TTM-21)

(2) They try to rationalize new information and integrate it with preexisting mental modes to reduce cognitive dissonance, seek cognitive closure, and circumvent potential breakup. For example, they will adopt the idol’s perspective to rationalize their behavior, believing that the idol should always be supported no matter what happened, and make self-adjustments to maintain commitment to them:

He’s not an idol, and it’s normal to be in a relationship. (B-db-JDS-19)

However, for behavior that cannot be rationalized, they will adopt criticism, apology, and other ways to accept them:

Take the criticism… learn from it… apologize for him… (B-wb-ZSQ-21)

(3) They overturn the preexisting mental modes and believe that the idol has behaved negatively, or gone against their own imagination and trust, or breached the bottom line of social ethics and the rule of law by violating the psychological contract with fans, resulting in the identification and legitimacy of breakup:

Don’t lie to your fans that you are single while you are secretly in a relationship. This is the most shameless. (B-wb-FLO-21)

During recovery, one should make the right decision instead of continuing to love him mindlessly. (B-wb-YYL-21)

In this phase, the three cognitive frameworks may emerge in different sequences. The fans switch repeatedly between the states of “leaving” and “returning,” gradually moving forward to the final decision:

A lot of people won’t explicitly say they want to leave now but wait a month, you will see, they’ll slowly run away. (A-YGD-20)

I’ll never quit idolatry and revenge. Never!… But I really need to take a break… I feel so terrible. I thought I didn’t care anymore, but I still cried… (A-MAR-20)

I feel like I did quit, yet I feel I didn’t. I deleted all the photos yesterday, but I woke up this morning and tried to restore them all… It seems I can’t stop loving him… Once I turn on my mobile phone, it’s still all about him. (B-wb-MXH-21)

Quitting is actually a long process. Countless times the thoughts arise and are suppressed until you finally face the reality… (B-db-KAD-18)

Before negative events occur, fans have already developed strong emotional attachments and trust in their idols which are highly associated with behavioral dependence (Rojek, 2006; Xie, 2021). Therefore, in the bargaining phase, in addition to adjusting their cognition, fans need to weaken emotional and behavioral dependencies. Such emotional attachment and behavioral dependence create obstacles to the psychological adaptation process:

Loving you has become my habit, a habit I can’t break for the rest of my life. (B-wb-NYB-20)

I’m a person, not a thing. It’s not that easy to walk away after two years of love. (C-EVA-20)

Finally, fans must detach from both the idol and the fan community. They may face social and moral criticism from other fans and the risk of cyber-violence. For example, the remaining fans may attack the exiting fans:

She climbed the wall,^3^ which made me sick… We unfollowed each other a long time ago. (A-BQS-20)

Those who quit and revenge are so ill-bred. How can you do this to the idols you really liked before? (B-wb-DHD-20)

I really can’t understand those who repeatedly quit and come back. Would a normal person with good mental health do that? (B-wb-DYY-20)

Other fans express their concerns about exiting the community:

I actually admire you,^4^ but I don’t have enough courage. I’m not as brave as you. (C-HTX-20)

In particular, traditional Chinese culture emphasizes collective interests and interpersonal dependence, leading to loneliness being less accepted (Cheng et al., 2011). The decision to disengage from the community can trigger strong social anxiety in individuals, making it an important factor preventing fans from exiting (Erath et al., 2007).

Some fans fail to overcome obstacles in the negotiation phase, including failing to overturn the preexisting mental modes, weaken emotional attachment, attenuate behavioral dependency, or cope with fear of cyber-violence from other fans. They are therefore unable to enter the recovery phase and continue to adopt the first or second type of cognitive framework and remain in the attachment state, that is, failing to quit or returning:

I thought there would be no more fans left in this group, but still many of them came back. (A-ADJ-20)

Although some fans do not ultimately quit the community, they are able to achieve growth through trauma recovery, and their mental modes and behavioral habits may change greatly: for example, changing from the unconditionally loyal type to bounded loyal type or short-term rational type:

I won’t use this account anymore, and I’m going to be a happy passerby fan again. (B-wb-XYT-21)

*Proposition 1B:* In the negotiation phase, fans need to overcome four types of obstacles: cognitive dissonance, emotional attachment, behavioral dependence, and social threats.

#### Attachment reconstruction – Recovery phase

Recovery is the process of regaining the social, psychological, and physical capacity for life and work. Studies have shown that individuals experience recovery in three main aspects: psychological, physical, and overall well-being improvement (Pizarro, 2004). In the late stage of the renouncement process, from a physical aspect, somatization symptoms gradually disappear; from a psychological aspect, negative emotions gradually weaken, and deep identification with and attachment to idols and communities are eliminated; from a behavioral aspect, the dependence on mobile phones to chase the star is lessened. However, they still show high sensitivity to environmental cues, and require a longer period than the first two phases to complete their psychological adjustment to achieve post-traumatic growth:

Once I broke free from idolatry… I also felt like my bones and blood were taken away, and I needed to take great care of myself to recover slowly and avoid reading any information that might hurt my feelings. (B-wb-QK-21)

[After continuing to fight for him these days,] I am too sensitive and weak right now … (B-wb-NT-19)

It took me three days to calm down and slowly recover, and it took me about half a month to fully recover. (B-wb-JX-21)

This study found that there were two main strategies for fans to recover from the loveshock. The first is healing the past, which includes the following actions: (1) *Engage in public outcry*, such as carrying out retaliatory aggression to balance the sense of relative deprivation (e.g., revealing idol’s secret and performing revenge, and expressing real anger against the idol and the fan community publicly). This kind of healing behavior is usually not accepted or recognized by the community, because it might harm the idol or the cohesion of community and is therefore not conducive to collective goals. However, from the perspective of fans who are renouncing fanship, suppressing negative emotions can lead to heavier trauma and self-destructive behavior and aggravate depression, making it less conducive to recovery from trauma. (2) *Share breakup plans.* Fans share their trauma experience and traumatized emotions with fellow renouncing fans to obtain social identity for leaving and relieve the psychological distress during the recovery phase:

I’ve been doing nothing but looking for people to talk to. ** said to rest for a few days first. ** also found it a bit hard to accept. *** seemed to be okay without being affected by anything. (C-YDR-20)

I agree with you. We definitely have the freedom to leave. They deserve it. (B-wb-XHY-20)

(3) *Reconstruct cognitive framework, reduce cognitive dissonance, and seek legitimacy for their recovery behaviors*:

I’m really tired of worrying about these stars… I’ve spent tens of thousands or even more chasing each of them… the results were all bad, so now my principle is not to spend a penny, whoever it is. (B-wb-GUE-21)

(4) *Seek closure to the virtual role as a super fan.* Fans make an active decision to stop idolatry, weaken their community dependency, and psychologically exit the fan role. Some fans adopt a flexible strategy to detach from the community by explaining their decision and saying goodbye:

I’ll spend less time on Weibo from now on, simply because I have to take exams… I just need to concentrate on studying, please forgive me. (B-wb-MEL-20)

Some fans use a more direct strategy to express a clear attitude, publicly declaring that they are no longer a fan and about to leave the community. In this case, they will have no chance to “return” and must complete the breakup process. For example, they may publicly post on Weibo, unfollow each other, change their avatars, IDs, or accounts, and delete posts and photos related to the idols.

The second strategy is to face the future, which includes the following actions: (1) *Switch the environment, moving away from the traumatic environment and cues.* According to attention restoration theory, fans’ excessive addiction to social media and fan groups brings directed attention fatigue, making them irritable, impatient, and unable to focus (Ohly et al., 2016). Previous studies suggest that remaining in risky environments for prolonged periods can easily lead individuals to be involved in high-risk behavior patterns. Fans are prone to develop unhealthy interpersonal relationships and engage in high-risk behaviors in a fan group with a tendency for deviant behaviors (Griffiths, 2001; Griffiths, 2010). Therefore, fans need to switch to a healthier environment in the recovery phase. In a restorative environment, directed attention fatigue can be effectively relieved, positive emotions can be increased, negative emotions can be reduced, and psychological stress can be relieved, leading to the achievement of psychological recovery (Korpela et al., 2008). For example:

I’ll focus on work and study for the next two months. (B-wb-ZQQ-21)

The holiday is coming soon, and I plan to go out to get rid of these annoying things [e.g., fandom conflicts]. (C-SNC-20)

(2) *Shift attention to other interests, explore new pleasures and seek to establish new recreational style*s:

The fan leader ** now… posts news about the Olympics and the pandemic after a successful transition. (B-db-FCZ-21)

I unfollowed her a long time ago. She is a fan of the new top star now. (A-BQS-20)

I see that she is posting about food and parties on Weibo from morning to night now. (C-DXJ-19)

(3) *Inhibit intrusive rumination, gain experience in coping with trauma, and remain in an equanimous state*:

I’m so sad, after all I truly liked him… before… I’ve quit idolatry… forget it, that’s it. Whatever it is, I don’t care anymore. (B-wb-CPT-20)

*Proposition 1C:* In the recovery phase, strategies can be divided into two categories: healing the past and facing the future.

### Factors influencing the renouncing process

Studies have shown that there are large individual differences in the trauma recovery process. For example, some fans indicated that:

There are sunk costs. Some people accept it quickly, some people recover slowly… (B-wb-ZLW-21)

This study investigated the factors that influence the recovery process from breakup and loveshock and summarized the internal and external factors.

The internal factors are as follows: (1) *The level of initiative*. Previous studies have shown that after the breakup of an intimate relationship, the emotional and behavioral reactions of the active and passive partners differ. The active partner who initiates the breakup tends to feel relaxed and guilty while the passive partner loses their autonomy to control the relationship and is more likely to experience anger, confusion, insecurity and strong emotional reactions (Cohen, 2004). Similar to romantic breakups, fans also showed active and passive exits. Active breakup means that a fan takes the initiative to stop idol worshipping and decides to quit after sufficient consideration. Passive breakup refers to a fan who was in a state of deep attachment but was impelled to stop being a fan after being exposed to the traumatic events. Like the passive partners in breakups who are more likely to undergo a psychological crisis, fans who passively quit idolatry also experience stronger traumatic stress reactions and have a longer recovery process. Fans who actively quit idolatry are more resolute in their attitudinal changes, less tolerant of negative events, and are more psychologically prepared to renounce idolatry. For example, short-term rational fans might “suddenly become indifferent, just because of small things” (B-db-TRM-20), and short-term passionate fans might say “It’s a matter of principle. Any explanation is useless. Anyway, I’m absolutely quitting” (B-wb-ZGF-21). Meanwhile, passive breakup is usually the result of primary and secondary trauma caused by negative events concerning the idol or fan community. Fans are usually not adequately prepared to deal with the trauma. Their core belief systems are broken, and they have difficulty in accepting reality:

Every day during this period of time, I pick up my phone and fight the anti-fans. Why is everyone attacking him? I really can’t hold on anymore. (C-XBA-18)

Passive breakup can also transform into active breakup:

I have realized that there’s something wrong with my emotions, and I am trying to save myself. (B-wb-ZLW-21)

(2) *Attribution style.* Fans usually create internal attributions for idols’ successes and strengths and adopt external attributions for negative events concerning idols. Similarly, fans usually create internal attributions when group behavior is identified with and adopt external attributions when group behavior is criticized. When fans are accustomed to creating external excuses for negative events, they generate more feelings of relative deprivation and perceived social injustice. This stimulates more negative emotions and resistant or attacking behaviors, and they are more inclined to adhere to the preexisting mental modes in the negotiation phase, making it more difficult to enter the recovery process. Fans who can make internal attributions for negative events are more likely to experience active breakup and shorter recovery periods.

(3) *Trauma recovery experience and fandom engagement experience.* This study found that fans who had multiple experiences of breaking up with their idols or had admired multiple idols showed more dominant behaviors in the group and were less likely to be dominated, mostly with conditional and bounded attachment to their idols. Emotional and rational orientation coexisted in their star chasing behavior and they had more diverse coping strategies and greater autonomy when recovering from idolatry, but they were also more likely to develop deviant behaviors:

I don’t care about the exposure to romantic relationships. Every idol may fall in love. I’m used to it. If I don’t like the idol anymore, I’ll find another one. There is no shortage of handsome men and beautiful women in the entertainment industry. (A-YGD-19)

Individuals who are younger with higher social anxiety or lack fandom experiences are more likely to be held hostage by the group to generate unconditional attachment, and invest more emotional labor, and material capital. Therefore, their trauma is more serious when experiencing negative events, and they have longer recovery periods with a tendency to repeatedly shift between “returning” and “leaving,” making it difficult to complete the psychological adjustment of recovery:

It’s the first time I’ve ever been such a fan of a star. It never happened before, and it probably will not happen again in the future… I’m too sad, and I don’t know how long it will last… but I’ll really feel sad and disgusting if I don’t quit idolatry. (C-GHN-19)

(4) *Attachment status and core belief system*. When fans adopt a core belief system of unconditional trust and full attachment, they over-invest in fan capital (both tangible and intangible), resulting in significant sunk costs, and thus they are more likely to experience psychological crises, stress reactions, and difficulty in recovering from psychological trauma when the psychological contract breaks down:

She had been operating this fan site for six years and spent hundreds of thousand yuan on him. No wonder she gets so pissed off. I would also seek revenge if it was me. No one can put up with this. (B-wb-WDR-20)

You know how much I loved him. I fought hard for his data even when my grandma was ill. I just feel so stupid right now. Every time I see his face or think of his name, I feel like throwing up. (C-YHJ-20)

(5) *Alternative lifestyles*, which mainly refer to the ease with which fans can change their behavioral pattern, and whether they can adopt more positive and diverse coping strategies to replace idolatrous behaviors and online dependence:

I have been watching this TV show these days. There are so many fun things out there. I immediately run away if you cannot make me happy any more. (B-wb-QLK-20)

I don’t know what else to do. I’ve already get so used to the fan life. It’s hard to change that. (C-HTX-20)

Fans who have diverse pleasures in life are more likely to withdraw attention from idols and can use a variety of means to relieve the discomfort of trauma recovery, such as exercising, socializing, traveling, or watching movies. There are also fans who simply switch their idol of choice without reducing idolatrous behavior:

It’s normal that these fans go back and forth. There are so many new idols out there if they want to follow. (A-YGD-20)

*Proposition 2A:* The major internal factors that influence adaptation include the level of initiative, attribution style, previous experience, core belief system, and alternative lifestyle.

External factors mainly include the following: (1) *Social support*. Fan behavior is often not accepted or approved of, and therefore it is also difficult to receive understanding from family and friends when traumatic stress reactions occur. A “fan” is often regarded as emotional, extreme, irrational, and unable to distinguish right from wrong:

His fangirls and fanboys are just like being brainwashed in a cult, with no brain left. (B-wb-WTE-20)

Why do you have to do this for a star? Does the star know you? (B-db-YHT-20)

Therefore, fans often experience a lack of support when dealing with traumatic stress responses concerning their idols. Their stressful response might even invite aversion and revulsion from non-fans, friends, and family, which triggers deviant behavior and prolongs the recovery time from trauma. This leads to negative spillover effects, affecting the formation of their character and values. (2) *Peer pressure from the fan group*. The more open and inclusive a fan group is, the more support and understanding fans receive when renouncing the idol and the community and the faster they can recover from trauma:

The idol has not produced any work in two years and did not take care of [their] fans. The fans have long wanted to run away, which is understandable. (A-ADJ-20)

The more cohesive the fan base, the higher the dependency and loyalty of fans, and the less likely the fans are to disengage:

I thought I’d persist a month at most, but it’s been two years. Without **, I probably would have run a long time ago. (A-BQS-20)

Meanwhile, an exploitation-oriented fan base with a stronger transgressive atmosphere and a more destructive leadership style can lead to increased mental stress, depleted psychological resources, and greater difficulty in recovering from the para-loveshock of breakup:

That group was downright toxic, and it almost made me quit idolatry. As soon as I realized it, I disengaged from the group. (C-HTX-19)

(3) *External life stress.* Previous studies have shown that boredom and free time are important predisposing factors that spawn Internet addiction, social media overuse, and idolatrous behavior in students when there is a lack of external stressors from schooling, work, and family. External stressors can stimulate mental resilience in individuals, making them focus on overcoming real-life difficulties and thus forgetting about trauma in the virtual world:

My son complains that I only care about the idols but not him. Family is still the most important. (A-ANM-18)

(4) *Types and impact of traumatic events*. There are different types of events that cause fans trauma with different levels of impact, ranging from small verbal conflicts between individuals to idols breaking legal and moral boundaries. The more destructive the traumatic event is to public order and morality, the wider the scope of impact, and the stronger the fermenting effect of public opinion, the more severe the primary trauma and the more lasting the secondary trauma suffered by fans:

Can you imagine the idol that you have been supporting with all your heart committing so many disgusting crimes? His fans all collapsed! Now I think it’s a lucky thing that our idol only has romantic scandals … (B-db-NYT-21)

Long-term engagement in the strife of fandom can cause fans to experience frequent psychological crises, making it difficult for them to adjust and recover:

His fans are so poor. They keep fighting for him online everywhere for almost a year after he was banned. They are obviously desperate and out of their mind right now. (A-XRW-21)

I would never ever set foot in any fan community again. There is only endless fighting, compulsive buying, and free labor in there. Nobody treats you like a person. (C-CYD-19).

*Proposition 2B:* The major external factors that influence adaptation include social support, peer pressure, life stress, type and impact of traumatic event.

### Types of fans and corresponding breakup style

Breakup style can be highly associated with attachment style. Therefore, we tried to observe the fans on two dimensions reflecting attachment status to the idol before breakup (orientation and commitment) and classified fans into four types: short-term rational, short-term passionate, bounded loyal, and unconditionally loyal fans. Orientation mainly reflects the level of fans’ emotional attachment with their idols. Emotion-oriented fans focus on the emotional experience in the process of chasing stars, with a higher level of emotional attachment to idols and emotional arousal. Rational-oriented fans have a lower level of emotional attachment to their idols and lower emotional volatility. The dimension of commitment mainly refers to the level of sustained loyalty, attachment, and identification of fans with their idols and groups. Fans with low levels of commitment prioritize their interests in the real world and capitulate and sacrifice the interests of idols and communities first when their personal interests are in conflict. With more attention to their own real-world interests, they have lower attachment to communities and idols. In contrast, fans with high levels of commitment pay more attention to their virtual roles than their real-world roles and sacrifice their own interests for the interests of idols and fan communities. While the internal factors in previous section focused on the process after breakup and determined how fast the fans can leave their idol, this section focuses on the process before breakup and reflects how much the fans loved their idol.

*Proposition 3A:* Fans can be classified into four types according to their attachment status on two dimensions: orientation and commitment.

This study classified fans with low commitment levels and rational-oriented characteristics as short-term rational fans, similar to terms used in fandom such as “fan for three months,” “passerby fan,” and “passing liker.” These fans usually have low levels of addiction and attachment, maintain conditional identification and trust, with loose idol-fan *quid pro quo* and short durations of emotional energy. Therefore, they have a low level of stress, recover quickly from trauma when renouncing fanship, and belong to the non-traumatic breakup style. Emotion-oriented fans with low levels of commitment could be classified as short-term passionate fans, with high levels of emotional attachment, and strong appeal and cohesion. However, correspondingly, their expectations of idol-fan *quid pro quo* are higher, and thus they are more prone to negative emotions and perceived injustice and have stronger stress responses when the psychological contract is violated, leading to shorter duration of attachment. This may manifest in voicing opinions, exiting, revenge, “climbing the wall,” “fighting,” or other behaviors belonging to the stressful breakup style. Bounded loyal fans have low levels of emotional volatility and low emotional investment and are better able to distinguish between online fan roles and offline real-world roles, with lower exchange requirements for idols and communities. Their core belief system is conditional trust and identification, that is, they perceive the negative behaviors of idols and communities more objectively as they do not hold high expectations from the very beginning. Therefore, they are less likely to have psychological contract breakdowns and can become long-term loyal fans instead. Their quitting decision usually forms over time. Unconditionally loyal fans refer to fans with high levels of emotional investment and commitment to idols and groups, similar to the label of “die-hard fans.” They possess unconditional trust in and complete attachment to their idols, are deeply involved in and lead support activities, remain loyal for a long time, and have high levels of emotional stability with certain mental resilience. Therefore, they may also experience the strongest traumatic breakup due to such deep involvement. The classification of fans with their corresponding breakup style is shown in Table 3.

**Table 3:** Classification of fans and breakup style.

	Rational-oriented	Emotional-oriented
Low levels of commitment	Short-term rational fans non-traumatic breakup	Short-term passionate fans Stressful breakup
High levels of commitment	Bounded loyal fans accumulated breakup	Unconditionally loyal fans traumatic breakup

*Proposition 3B:* Based on the four types of fans, there are also four corresponding types of breakup style.

### Post-breakup growth of fans

Appropriate stress responses enable individuals to adapt to environmental changes and develop their character. Positive psychological changes can be achieved during coping with life crises and overcoming traumatic experiences, that is, post-traumatic growth (Tedeschi and Calhoun, 1996). Previous research suggests that post-traumatic growth may manifest itself in terms of altered interpersonal experiences, altered self-awareness, and altered life values, and can be an opportunity to promote individual development and achieve self-transcendence (McLean et al., 2013; Ramos and Leal, 2013). This study indicated that the post-renouncement growth of fans was reflected in four main aspects: mental modes, coping skills, emotional adaptation, and behavioral patterns toward para-loveshock. Growth of mental modes mainly refers to fans’ fresh understanding of the relationships between themselves, their companions, groups, and idols through the traumatic events:

I had been scolded countless times by both outsiders and insiders when I was in the fandom. I was attacked, misunderstood, cursed, and excluded. Now I can talk with a smile about the people and things that I hated in the fandom. I’ve grown up. (B-wb-ZC-20731)

I regret the money I used to spend on chasing stars. It’s not easy to earn money! I must have been out of my mind before! (B-wb-JNL-21)

Eventually, the relationship between friends in the fandom ends. There are no eternal friends made by chasing stars in the virtual world. (C-ADJ-20)

The second is the growth in trauma coping skills. Fans acquire experiences of coping with attachment breakdowns, core belief violations, and stress responses as they engage in idolatrous behaviors over time and can respond to negative events in more positive, varied, or skilled ways to recover faster:

The idol’s image has fallen too many times, and no wonder. (A-HWD-21)

Don’t get emotionally involved when you’re chasing a star! Don’t get too attached! (B-db-AMY-21)

The third is the growth in emotional adaptation. The fans’ emotional sustenance changes:

Is there anyone in this group like me who becomes indifferent to the stars… the fan community is messy… only the person who inspires me deserves to be called an idol. (B-db-WQP-21)

The ones who get excited are the new fans, the old ones are not surprised at all. (A-HJ-20)

The fourth is the growth in behavioral patterns. They find that the past behavioral patterns are no longer appealing or want to change their negative behavioral patterns. For example, they are no longer addicted to mobile phones or social media; they deny or even criticize fan behavior, and express remorse and shame for their past behaviors. This is followed by finding, changing, and building new patterns of life and behavior, such as returning to real life (e.g., studying, working, exercising, and socializing), or seeking other pleasures (e.g., playing games, watching videos, shopping).

*Proposition 4:* After psychological adaptation, fans may show post-breakup growth in four aspects: mental modes, coping skills, emotional adaptation, and behavioral patterns toward para-loveshock.

## Conclusions and discussion

### Research findings

**Figure 1:**
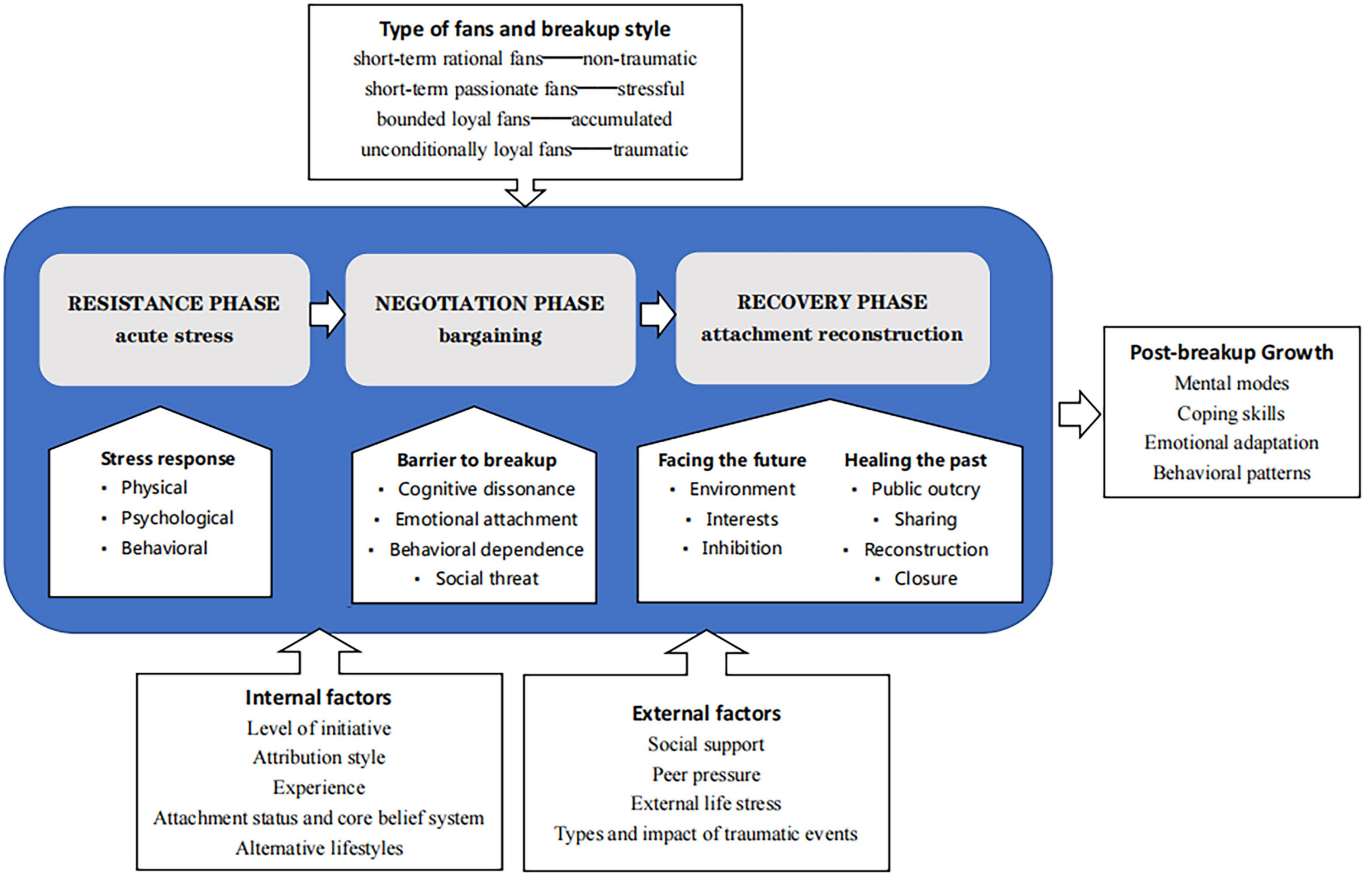
The integrated theoretical model of this study.

In this study, we divided the process of renouncing fanship into three phases: the resistance phase with acute stress, the negotiation phase with bargaining, and the recovery phase of attachment reconstruction. In the resistance phase, fans experience acute stress in physiological, psychological, and behavioral respects. In the negotiation phase, fans face four barriers to get past breakup: cognitive dissonance, emotional attachment, behavioral dependence, social threat, and bargaining between three types of cognitive frameworks (adherence to the preexisting mental modes, rationalization and integration of new information, and overturning the preexisting mental modes) to decide between leaving or returning. In the recovery phase, the stress response is reduced, and fans promote recovery through strategies of healing the past and facing the future. Healing the past mainly refers to public outcry, sharing breakup plans, cognitive reconstruction, and seeking closure to the online fan role. Facing the future includes switching the environment, shifting attention to new interests, and inhibiting the re-intrusion of trauma cues. The three phases of psychological adaptation may overlap, and the boundaries may be blurred, indicating some individual differences; for example, fans may have already begun to adopt recovery strategies when bargaining. Overall, the resistance phase reflects the acute stress response due to disbelief/shock happening within the first several hours or days, which is obviously a downturn; the negotiation phase reflects hesitation, struggling, and strong effort to persuade themselves in the following days; and the recovery phase reflects the great effort they make to seek help for a gradual upturn, which may take weeks or even months. The internal factors that influence the psychological adaptation process include the level of initiative, attribution style, experience, attachment status and core belief systems, and alternative lifestyles. External factors include social support, peer pressure from the fan group, real-life stress, and types and impact of traumatic events. According to the two dimensions of orientation and commitment, fans can be broadly classified into four types: short-term rational fans, short-term passionate fans, bounded loyal fans, and unconditionally loyal fans, respectively corresponding to non-traumatic breakup, stressful breakup, accumulated breakup, and traumatic breakup behaviors. The post-renouncement growth of fans mainly manifests as growth in mental modes toward trauma, trauma coping skills, emotional adaptation experience, and improved behavior patterns. According to our findings summarized above, we built an integrated theoretical model as illustrated in Figure 1.

### Discussion

The phase delineation in this study drew from established theoretical models. According to the current data, we mainly referred to the five-stage and four-stage theories of grief, and general adaptation syndrome, simplifying our model into three phases (Kübler-Ross, 1973). The five-stage model of grief, including denial, anger, bargaining, depression, and acceptance, has been used to study the loss of a relationship, the death of an important person, and responses in a time of crisis (Bolden, 2007; Stephens et al., 2021). The four-stage model includes numbness-disbelief, separation distress (yearning-anger-anxiety), depression-mourning, and recovery (Jacobs, 1993). General adaptation syndrome is described as three stages: alarm reaction, resistance, and exhaustion (Selye, 1950). By comparing the denial, anger, alarm, and resistance stages, it was determined that “resistance” would serve better in reflecting the first stage of fan responses. This phase was followed by the “bargaining” and “negotiation” phase which is considered a common and inevitable step in many existing models. Finally, “recovery” was used to refer to the long progress that covers more than depression and acceptance after a final decision was made. Among the three phases, the recovery phase should occupy the longest period (up to weeks or months for some fans), while the first and second phases usually take several hours to a few days. Some stages in other models were already covered by our three phases: for example, alarm reactions, physiological complaints, seeking comfort, cognitive restructuring, rational attribution, emotional expression, and reinvestment. Overall, there are similarities and differences between our study’s model and previous ones. The similarities lie in the experiences of anxiety, tension, resistance, wavering, and then moving to self-adjustment and acceptance. The difference lies in the fact that romantic breakup and bereavement is a two-way intimacy rupture, while the intimacy in idolatry comes from parasocial interaction and community attachment. It is a one-way mimetic intimacy, an intimacy that is built in virtual space and imagination, which can exist only from the perspective of the fans. Renouncing fanship is more of a psychological withdrawal of attachment, while the idol is not involved in person, and the fan can take control of the one-way attachment, that is, one-way breakup or one-way “re-following.” Therefore, the psychological adaptation process of breakup with the idol is different from that with an intimate partner, or other loss of intimacy such as bereavement.

Pre-trauma risk factors typically include gender, personality, and age. For example, individuals with high psychological capital are better able to cope with traumatic events and have access to higher social support and coping skills. Peri-trauma risk factors include the degree of traumatic exposure, subjective fear, and core belief challenges. Coping style, rumination, resilience, sense of control, social support, and gratitude were found to be post-trauma risk factors (Sayed et al., 2015). Although loveshock recovery is similar to trauma recovery, there are certain differences. This study suggests that fans are faced with four aspects of hindrances during breakup with the idol: cognitive dissonance, emotional attachment, behavioral dependencies, and social threats. Based on these obstacles, we summarized the common factors that influence fans’ post-breakup psychological adaptation, including several factors such as initiatives, attributional styles, experiences, alternative lifestyles, peer pressure, life stress, and types of traumatic events, in addition to the core beliefs and social support mentioned in previous studies. On this basis, this study also classified the fans and their breakup process into four types, which extends the theory of idolatry and stress recovery.

### Implications for practice

Based on the findings of this study, we suggest the following to address the psychological adaptation process of fans’ para-loveshock due to renouncing an idol. First, fans should not only learn how to “love” the idol, but also how to break up with them, that is, to grasp the coping skills that enhance post-breakup recovery efficacy, accelerate the psychological adaptation process, reduce the negative impact from loveshock, and promote post-renouncement growth and psychological resilience. Fans can identify the categories and phases they belong to according to our research, and adopt corresponding strategies to relieve stress responses, including: (1) when encountering negative events as a fan, one should be allowed to express real feelings and talk openly to balance the perceived relative deprivation and injustice. The fans need to keep away from the emotional manipulation and admonishment of “fandom culture.” In this way, self-aggression and depression can be effectively reduced, and negative spillover effects from the fandom can be avoided; (2) Fans should perceive and accept the negative information in an objective way instead of refusing it without thinking so that the first and second phases can be shortened. By changing non-adaptive cognition and attributional styles, fans can break the “divine” fantasy about idols and accept the “human” reality of them, stop the aggressive, exclusionary, and exploitative mindset of the “fandom culture,” and avoid falling into love addiction, smartphone addiction, and group polarization and groupthink on social media; (3) After deciding to quit, the fans should stick to the determination, go public with the renouncement, and seek a firm closure to the social identity of a fan, that is, stop fighting for the idol any more to avoid first and secondary trauma; (4) To facilitate the recovery phase, fans should disengage from the online environment, reform lifestyle and behavior patterns, reduce mobile phone and social media use, keep away from traumatic cues, improve offline social activities, and develop new pleasures and hobbies; (5) One should stay away from fellow fans and fan groups that have deviant behavioral tendencies and spread negative emotions, be alert to the compulsive and destructive atmosphere of the group, and protect personal privacy from cyber-violence.

Second, from a social perspective, it is necessary to cope with the chaos of “fandom culture” by destigmatizing fan behavior in a broad sense and preventing more impulsive and deviant fan behaviors. The deeper the involvement of fan behavior, the heavier the negative events experienced, the more sensitive the individual fans are to negative environmental stimuli, and the more severe the psychological trauma they may suffer. Therefore, family members and friends should pay more attention to the psychological crises of deeply involved fans, demonstrate some understanding, and offer support to fans who show high levels of stress responses, instead of engaging in controlling or abusive treatment, thereby helping them complete trauma recovery and achieve post-breakup growth.

### Limitations and suggestions for the future

This study has several limitations. First, due to the numerous data that constantly emerged, it was impossible to collect and store all the relevant material from all fans in a community. Therefore, the conclusions may require further quantitative validation. In the future, big data can be extracted and machine learning adopted to explore the psychological and behavioral aspects. Questionnaires can also be used as measurement tools to collect quantitative data and validate our research findings. Experiments with measurement of physiological indicators will be another contributing approach to obtain objective data. Another limitation is that the researcher mainly focused on the fan community of a certain typical young pop idol. Although our data was not limited to that idol and we also focused on negative events that triggered extensive losses of fans from other idols, most of the semi-private data was obtained from the fan community of one single pop idol. However, we do not consider this to have significantly biased our results because the triangulation validation with multiple sources of data verified our conclusions in many fan communities. In the future, other fan communities can be investigated to explore potential heterogeneity and advance our theoretical model. The third suggestion lies in the age cohort of fans. The current study mainly focused on fandom fans whose age range is mostly below 40 years old. However, due to the high prevalence and accessibility of smartphone and social media platform, the age range and number of fans is expanding fast in China. There might be a large number of middle-aged and elderly fans who are not deeply involved or active in online fan community and are deemed as passerby fan, passing likers, or occasional fans. Their star worship behavior should obtain equal attention. Although it was indicated that breakup shock may generate more crises for young, especially adolescent fans, it is proposed that the current conclusion can apply to deeply involved fans of all ages. As a result, further empirical evidence from other populations is needed to improve the external validity of our conclusion. The fourth limitation is that this study mainly emphasized on the individual level. Discussion on group level is not enough and requires further organization systematically. Thus, explanations related to the community should be a significant theme to consider. In the future, we could explore how fandom influences breakup behavior and mental modes compared with “solo” fans, or what is the difference between a single breakup event and large-scale breakup events, etc. Despite these limitations, this qualitative study has provided sufficient evidence to reveal how passionate fans renounce their idols and companions, identified the associated factors and the developing phases of such processes, categorized fans and their breakup responses to explain individual differences, and highlighted the positive aspects of undergoing para-loveshock and the process of breakup.

## Data availability statement

The raw data supporting the conclusions of this article will be made available by the authors, without undue reservation.

## Ethics statement

The studies involving human participants were reviewed and approved by Tianjin University. Written informed consent for participation was not required for this study in accordance with the national legislation and the institutional requirements.

## Author contributions

YH conducted the research and wrote the manuscript. YS contributed to the data analysis and article writing. All authors contributed to the article and approved the submitted version.

## Funding

This study was supported by grants from the post-funded project of National Social Science Fund of China for YH (21FGLB068).

## Conflict of interest

The authors declare that the research was conducted in the absence of any commercial or financial relationships that could be construed as a potential conflict of interest.

## Publisher’s note

All claims expressed in this article are solely those of the authors and do not necessarily represent those of their affiliated organizations, or those of the publisher, the editors and the reviewers. Any product that may be evaluated in this article, or claim that may be made by its manufacturer, is not guaranteed or endorsed by the publisher.

## Footnotes

^1^The source codes were as follows: A = community chat transcripts; B = Weibo and Douban platform post, comments, and interactions. B-type data would also be marked as coming from wb (Weibo) or db (Douban); C = private chat and interview transcripts. Network ID codes were the first two initials, first two letters, or last two initials of the ID. There might be instances of fans changing IDs or deleting/hiding posts. Only the time of year was shown in the code owing to ethical concerns. Specific months and days were kept in raw evidence.

^2^"She" and "her" refers to the fan.

^3^"Climbing the wall" refers to a fan switching their idol of worship, and their social media account persona changes from being a fan of idol A to being a fan of idol B.

^4^"You" refers to the quitting fan.
